# *HER-2* and *EGFR* mRNA Expression and Its Relationship with Versican in Malignant Matrix-Producing Tumors of the Canine Mammary Gland

**DOI:** 10.1371/journal.pone.0160419

**Published:** 2016-08-04

**Authors:** Karine Araújo Damasceno, Enio Ferreira, Alessandra Estrela-Lima, Conrado de Oliveira Gamba, Fernanda Freitas Miranda, Mariana Rezende Alves, Rafael Malagoli Rocha, André Luís Branco de Barros, Geovanni Dantas Cassali

**Affiliations:** 1 Department of General Pathology, Biological Sciences Institute, Universidade Federal de Minas Gerais, Belo Horizonte, Mina Gerais, Brazil; 2 Laboratory of Experimental Pathology, Gonçalo Moniz Institute, Fundação Oswaldo Cruz, Salvador, Bahia, Brazil; 3 Department of Pathology and Clinics, School of Veterinary Medicine and Zootechny, Universidade Federal da Bahia, Salvador, Bahia, Brazil; 4 International Center of Research in Cancer (CIPE), A.C. Camargo Cancer Center, São Paulo, São Paulo, Brazil; 5 Department of Clinical and Toxicological Analyses, School of Pharmacy, Universidade Federal de Minas Gerais, Belo Horizonte, Minas Gerais, Brazil; University of Patras, GREECE

## Abstract

Versican expression promotes tumor growth by destabilizing focal cell contacts, thus impeding cell adhesion and facilitating cell migration. It not only presents or recruits molecules to the cell surface, but also modulates gene expression levels and coordinates complex signal pathways. Previously, we suggested that the interaction between versican and human epidermal growth factor receptors may be directly associated with tumor aggressiveness. Thus, the expression of *EGFR* and *HER-2* in these neoplasms may contribute to a better understanding of the progression mechanisms in malignant mammary tumors. The purpose of this study was to correlate the gene and protein expressions of EGFR and HER2 by RNA *In Situ* Hybridization (ISH) and immunohistochemistry (IHC), respectively, and their relationship with the versican expression in carcinomas in mixed tumors and carcinosarcomas of the canine mammary gland. The results revealed that *EGFR* mRNA expression showed a significant difference between *in situ* and invasive carcinomatous areas in low and high versican expression groups. Identical results were observed in *HER-2* mRNA expression. In immunohistochemistry analysis, neoplasms with low versican expression showed greater EGFR immunostaining in the *in situ* areas than in invasive areas, even as the group presenting high versican expression displayed greater EGFR and HER-2 staining in *in situ* areas. Significant *EGFR* and *HER-2* mRNA and protein expressions in *in situ* carcinomatous sites relative to invasive areas suggest that these molecules play a role during the early stages of tumor progression.

## Introduction

The tumor microenvironment not only responds to tumor epithelial cells and supports carcinogenesis, but actively contributes to tumor progression and metastasis [1]. In a recent review, Theocharis *et al*. identify a direct relationship between growth factor-mediated signaling and modulating extracellular matrix (ECM) components [2]. Also, some data have shown that human epidermal growth factor receptor, a family of tyrosine kinase receptors, expressed in normal tissues and in many types of cancer in humans [3] and canines [4, 5] and its downstream pathway regulates migration and tumor invasion by ECM components [6].

Versican, an ECM component, is a member of the large aggregating chondroitin sulfate proteoglycan (CSPG) family and has been implicated in tumor progression [7]. Versican expression promotes tumor growth by destabilizing focal cell contacts, thus impeding cell adhesion [8] and facilitating cell migration. It has been shown that the C-terminal domain of versican modulates epidermal growth factor receptors type 1 (EGFR) and β1- integrin linkage. Together, these molecules regulate cell proliferation [9] and migration of neoplastic cells in the stroma [10–12]. Conversely, V3 versican isoform directly or indirectly interferes with the interaction of the heterodimer between EGFR and epidermal growth factor receptors type 2 (HER-2) in MeWo melanoma cells. This phenomenon alters the ERK1/2 and p38 MAPK signalling pathways and regulates cell proliferation and migration [10]. It seems that versican not only presents or recruits molecules to the cell surface, but also modulates gene expression levels and coordinates complex signal pathways [13].

We previously observed that members of the human epidermal growth factor receptor family, which are cell surface receptors, display a higher expression in aggressive tumors (carcinosarcoma) that overexpress versican in the stroma adjacent to the invasive areas, suggesting that the interaction between versican and these receptors may be directly associated with tumor aggressiveness [14]. Thus, the expression of *EGFR* and *HER-2* mRNA in these neoplasms may contribute to a better understanding of the progression mechanisms in malignant mammary tumors.

In small animal clinical practice, matrix-producing mammary tumors are common and they are characterized by epithelial, myoepithelial, and mesenchymal proliferation [4, 15, 16]. Formerly known as malignant mixed tumors, carcinomas in mixed tumors (CMT) and carcinosarcomas [17] can arise from the malignant transformation of the components of benign mixed tumors [4, 16, 17]. Some authors have used the term “metaplastic carcinoma” to refer to canine carcinomas in mixed tumors and carcinosarcomas [18–20]. Morphologically, these histological types are similar to metaplastic carcinomas in women and suggest that they could serve as research models for tumor progression [4, 15]. Molecular changes regarding the process of neoplastic progression of these tumors still remain unclear.

Thus, the purpose of this study was to investigate gene and protein expressions of EGFR and HER2 and their relationship with the versican expression in CMTs and CSs of the canine mammary gland.

## Materials and Methods

### Selection of cases

Fifteen and eight samples of CMTs and CSs, respectively, were selected at the Comparative Pathology Laboratory (*Universidade Federal de Minas Gerais*) and Veterinary Pathology Laboratory (*Universidade Federal da Bahia*). Samples of mammary tumors were obtained from female dogs of any breed or age, intact or spayed. The animals were admitted to the Veterinary Hospital of the *Universidade Federal de Minas Gerais* (UFMG) and Veterinary Hospital of the *Universidade Federal da Bahia* (UFBA) and were subjected to mastectomy with or without adjuvant treatment, between 2005 and 2012. Before surgery, all animals had complete clinical examinations that included hematology and serum biochemistry. Moreover, the dogs underwent thoracic radiography to rule out distant metastasis at the time of diagnosis.

### Anatomopathological study

Clinical staging was conducted based on tumor size (T), neoplastic involvement of regional lymph nodes (N), and presence of distant metastases (M) according to the Tumor-Node-Metastasis (TNM) staging system established by the World Health Organization for canine mammary tumors (modified from [21]). These data were collected from the clinical, radiological, and pathological records of each animal.

Four-micrometer histological sections were prepared from selected blocks and stained using the hematoxylin-eosin method. The histological type was confirmed according to the standards proposed by World Health Organization [22] and the Consensus for the Diagnosis, Prognosis, and Treatment of Canine Mammary Tumors [23]. Malignant invasive epithelial components in CMTs and CSs were graded according to the Nottingham System [24], which included tubule formation, nuclear pleomorphism, and mitotic index.

The *in situ* areas were defined by observing epithelial cells in a tubular arrangement with the myoepithelial cell layer and basal membrane integrity shown by HE [23].

### Immunohistochemistry

The primary antibodies used in the immunohistochemical analysis were versican (1:50, clone 12C5, DSHB, Iowa, USA), EGFR (1:100, clone 31G7, Invitrogen, California, USA), and HER-2 (1:200, polyclonal, Dako, Glostrup, Denmark).

For this technique, 3-μm sections were cut from one representative block of each case and collected on gelatinised slides. Tissue sections were deparaffinized, rehydrated in a graded ethanol series, and subjected to heat-induced antigen retrieval (water bath at 98°C for 20 minutes) with a target retrieval solution (DAKO) at pH 6.0, except for the slides intended for versican and EGFR staining. For versican, enzymatic recovery was performed using 0.5 U/ml chondroitinase ABC (*Proteus vulgaris*; Sigma Chemicals) in 0.25 M Tris buffer (pH 8.0) with 0.18 M sodium chloride and 0.05% bovine serum albumin (BSA) at 37°C for 1 hour and 30 minutes. A 0.25 M Tris buffer solution (pH 8.0) with 0.1 M 6-amino-n-caproic acid and 5 mM benzamidine hydrochloride was used for 30 minutes to inhibit protease activity (adapted from [25]). Enzymatic recovery of EGFR was performed using HCl-diluted pepsin at 37°C for 30 minutes. All of the slides were incubated for 15 minutes in 3% hydrogen peroxide in methanol to block endogenous peroxidase activity. Subsequently, the slides were covered with 10% normal rabbit serum in phosphate buffered saline (PBS) for 10 minutes and then incubated with the primary antibody overnight at 4°C (versican, EGFR and HER-2). Next, polymerization was applied, with identification based on secondary antibodies (ADVANCE HRP—ready to use—DakoCytomation). Diaminobenzidine was used as the chromogen, and the sections were counterstained with Mayer's hematoxylin, hydrated, and mounted in a synthetic medium.

Negative controls were prepared by replacing the primary antibody with normal serum. Canine mammary tumors previously known to express HER-2 and tissue with abundant myxoid matrix expressing versican were used as positive controls. Skin was used as positive controls for EGFR.

### Immunohistochemical evaluation

Versican expression in the stroma of adjacent areas to normal mammary gland and benign epithelial, malignant *in situ*, and invasive epithelial proliferation was assessed by the semiquantitative scoring system proposed by Skandalis *et al*. [26] and Damasceno *et al*. [16], which includes (i) the overall percentage of positively stained tissue (0–100%) and (ii) staining intensity for proteoglycan using the following scales: (1) negative or very weak staining, (2) weak positive staining, (3) moderately positive staining, and (4) strongly positive staining. The versican expression level was then calculated by the product of the percentage (i; 0–100%) of positive staining and the intensity of the staining (ii; 1–4). Based on the final results of this evaluation, a median versican expression score was obtained for the CMT and CS (median = 240) invasive areas. Thus, two distinct groups were determined for each histological type: group 1 (G1) was represented by cases with values below the median and considered to have low versican expression, and group 2 (G2) was represented by cases with values equal to and above the median and considered to have versican overexpression [16].

EGFR and HER-2 expression was assessed in epithelial tumor cells using the scores defined according to the consensus of the American Society of Clinical Oncology and College of American Pathologists (ASCO/CAP; Wolff *et al*. [27]) and adapted from Bertagnolli *et al*. [4] as follows: (-), no staining; (1+), weak incomplete membrane staining in more than 10% of tumor cells; (2+), incomplete and/or weak-to-moderate membrane staining in more than 10% of tumor cells or complete and strong staining in less than 10% of tumor cells; and (3+), complete and strong staining in at least 10% tumor cells.

Only epithelial components and adjacent stroma were evaluated in this study. At least five fields for each of the normal epithelial cells, *in situ*, and invasive carcinomatous proliferation in CMTs and CSs were analyzed.

### RNA *in situ* hybridization method

RNA *in situ* hybridization method was performed as previously described with minor modifications [28]. The RNAscope (Advanced Cell Diagnostics, Inc., Hayward, California) approach was used in archival formalin-fixed, paraffin-embedded (FFPE) tissue to view *EGFR* and *HER-2* mRNA in individual cells through a probe design strategy and hybridization-based on a signal amplification system to amplify signals and suppress background (*EGFR*: the reference sequence, XM_533073.4; probe region, 725–1660; *HER-2*: the reference sequence, NM_001003217.1; probe region, 1585–2823).

FFPE tissues sections four micrometers thick was deparaffinized in xylene, followed by dehydration in an ethanol series. Then, tissue sections was incubated in a pre-treatment buffer maintained at a boiling temperature (100°C to 104°C) using a hot plate for 15 minutes, rinsed in deionized water, and immediately treated with a solution of pre-treatment 3, which consists of a protease enzyme at 40°C for 30 minutes in a HybEZ hybridization oven (Advanced Cell Diagnostics, Inc., Hayward, California). Thus, the tissue was able to be incubated with the target probes that lasted for 2 hours at 40°C in a HybEZ hybridization oven (Advanced Cell Diagnostics, Inc., Hayward, California). After each hybridization step, slides were washed with wash buffer two times at room temperature. Preamplifier and amplifier molecules was hybridized in each probe pairs. Chromogenic detection was performed using diaminobenzidine (DAB), followed by counterstaining with Gill´s hematoxylin.

Assays using archival FFPE specimens were typically performed in parallel with positive and negative controls to ensure interpretable results. The endogenous housekeeping gene was used as a positive control to assess both tissue RNA integrity and assay procedure, and a negative control was used to assess background signals.

Staining results were evaluated by examining tissue sections under a standard bright field microscope at 20–60X magnification and categorized into five scores: (0) No staining or less than 1 dot to every 10 cells (60X magnification), (1+) 1–3 dots/cell (visible at 20–60X magnification), (2+) 4–10 dots/cell. Very few dot clusters (visible at 20–60X magnification), (3+) >10 dots/cell. Less than 10% positive cells have dot clusters (visible at 20X magnification) and (4+) >10 dots/cell. More than 10% positive cells have dot clusters (visible at 20X magnification) adapted from manufacturer's guideline. Cytoplasmic staining was only observed in carcinomatous cells.

### Statistical analysis

Statistical analyses were performed using the Graph Pad Prism v.5 software (San Diego, CA). The difference in versican, EGFR, HER-2, and mRNA expression between *in situ* and invasive areas was assessed using the Wilcoxon signed-rank test, and a Mann–Whitney U test (nonparametric data) was used to assess whether the receptor expression differed between groups (with high and low versican expression). The data were also subjected to Spearman's rank correlation coefficients. The values were considered significant when p<0.05.

### Ethical aspects

All procedures were performed under the guidelines and with the approval of the Ethics Committee in Animal Experimentation (CETEA/UFMG), protocol 0053/11.

## Results

### Versican expression in peritumoral stroma

Female dogs classified in low and high versican expression groups presented a mean age of 8.6 years (ranging from 6 to 14 years) and 10.23 years (ranging from 5 to 15 years), respectively. The clinicopathological characteristics of the versican expression groups are listed in Table 1.

**Table 1:** Clinicopathological characteristics of canine mammary carcinomas in mixed tumors (CMTs) and carcinosarcomas (CSs).

Parameters	G1	G2
	n/total (%)	n/total (%)
**Mean age**	8,6	10,23
**Size**		
**≤ 3cm**	1/8 (12.5)	2/13 (15.38)
**3 < x ≤ 5 cm**	4/8 (50)	5/13 (38.46)
**> 5 cm**	3/8 (37.5)	6/13 (46.16)
**Lymph node**		
**Metastasis**		
**Negative**	6/7 (85.71)	12/13 (92.31)
**Positive**	1/7 (14.29)	1/13 (7.69)
**Pulmonary metastases**		
**Negative**	6/8 (75)	13/14 (92.86)
**Positive**	2/8 (25)	1/14 (7.14)
**Clinical staging**		
**I**	1/8 (12.5)	2/13 (15.38)
**II**	4/8 (50)	5/13 (38.46)
**III**	1/8 (12.5)	4/13 (30.77)
**IV**	0/8 (0)	1/13 (7.69)
**V**	2/8 (25)	1/13 (7.69)
**Histological grade**		
**I**	5/9 (55.56)	7/14 (50)
**II**	2/9 (22.22)	5/14 (35.71)
**III**	2/9 (22.22)	2/14 (14.29)

(G1 = low versican expression; G2 = high versican expression).

Proteoglycan versican immunoreactivity in areas adjacent to the *in situ* carcinomatous regions was less intense (median, 150.0) as compared to the areas adjacent to the invasive regions (median, 240.0).

To define the high- and low-dividing versican expression median cut-off, we determined the versican expression at the stromal site adjacent to invasive areas in histological types (CMT and CS). First, group 1 (G1) (low versican expression) and group 2 (G2) (high versican expression) were defined.

**Fig 1:**
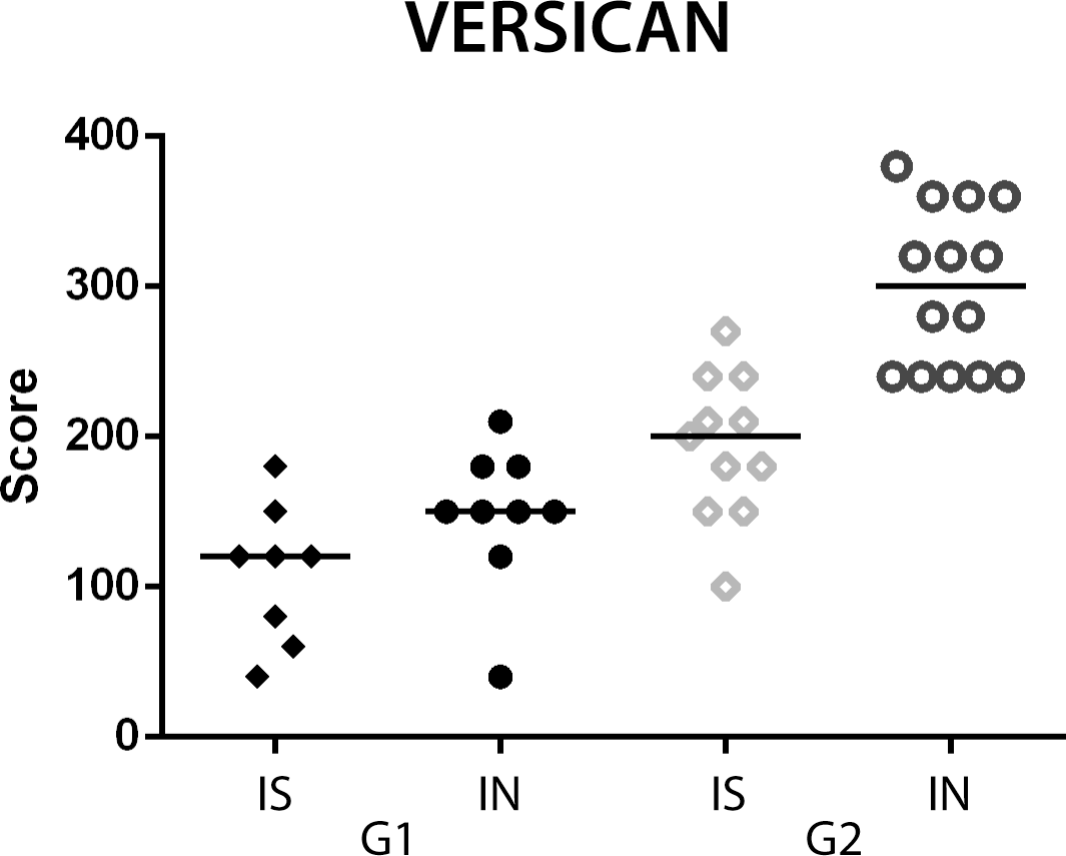
Differences versican expression in *in situ* (IS) and invasive (IN) carcinomatous areas in versican low (G1) and high (G2) expression groups in canine mammary tumours. Wilcoxon test.

Differences between the versican expression groups were evaluated and showed that the adjacent stroma in *in situ* carcinomatous areas revealed a lower versican expression in relation to the invasive areas in both G1 (*P* = 0.0078) and G2 (*P* = 0.0010) (Fig 1).

### *EGFR* and *HER-2* mRNA expression in carcinomatous areas

RNA-ISH was conducted in 23 cases, 15 CMTs and 8 CSs. No unspecific background staining was observed in any case. Nuclear dot-like staining was ignored for the final study as described by the manufacturer’s protocol.

Neoplasms showed different intensity levels as illustrated in Table 2. Normal mammary cells in the rest of the breast tissue were negative. *In situ* carcinomatous areas displayed scores 1 and 2 by *EGFR* mRNA expression analysis (both representing 33.33%) and score 1 by *HER-2* mRNA staining (38.89%) in the majority of cases. Most of the invasive carcinomatous cells presented score 1 for *EGFR* and *HER-2* mRNA expression (50% and 65% respectively) (Table 2).

**Table 2:** EGFR and HER-2 expression by immunohistochemistry (IHC) and their mRNA by *in situ* hybridization (ISH) in canine mammary carcinomas in mixed tumors and carcinosarcomas.

		IHC			ISH	
	Score	IS	IN	Score	IS	IN
		n/total (%)	n/total (%)		n/total (%)	n/total (%)
**EGFR**	0	0/21 (0%)	2/23 (8.7%)	0	2/21 (9.52%)	6/22 (27.27%)
	1	2/21 (9.52%)	10/23 (43.48%)	1	7/21 (33.33%)	11/22 (50%)
	2	5/21 (23.81%)	10/23 (43.48%)	2	7/21 (33.33%)	4/22 (18.18%)
	3	14/21 (66.67%)	1/23 (4.35%)	3	4/21 (19.05%)	1/22 (4.55%)
				4	1/21 (4.76%)	0/22 (0%)
**HER-2**	0	0/20 (0%)	1/23 (4.35%)	0	0/18 (0%)	4/20 (20%)
	1	2/20 (10%)	12/23 (52.17%)	1	7/18 (38.89%)	13/20 (65%)
	2	13/20 (65%)	9/23 (39.13%)	2	6/18 (33.33%)	2/20 (10%)
	3	5/20 (25%)	1/23 (4.35%)	3	2/18 (11.11%)	1/20 (5%)
				4	3/18 (16.67%)	0/20 (0%)

IS = in situ; IN = Invasive

**Fig 2:**
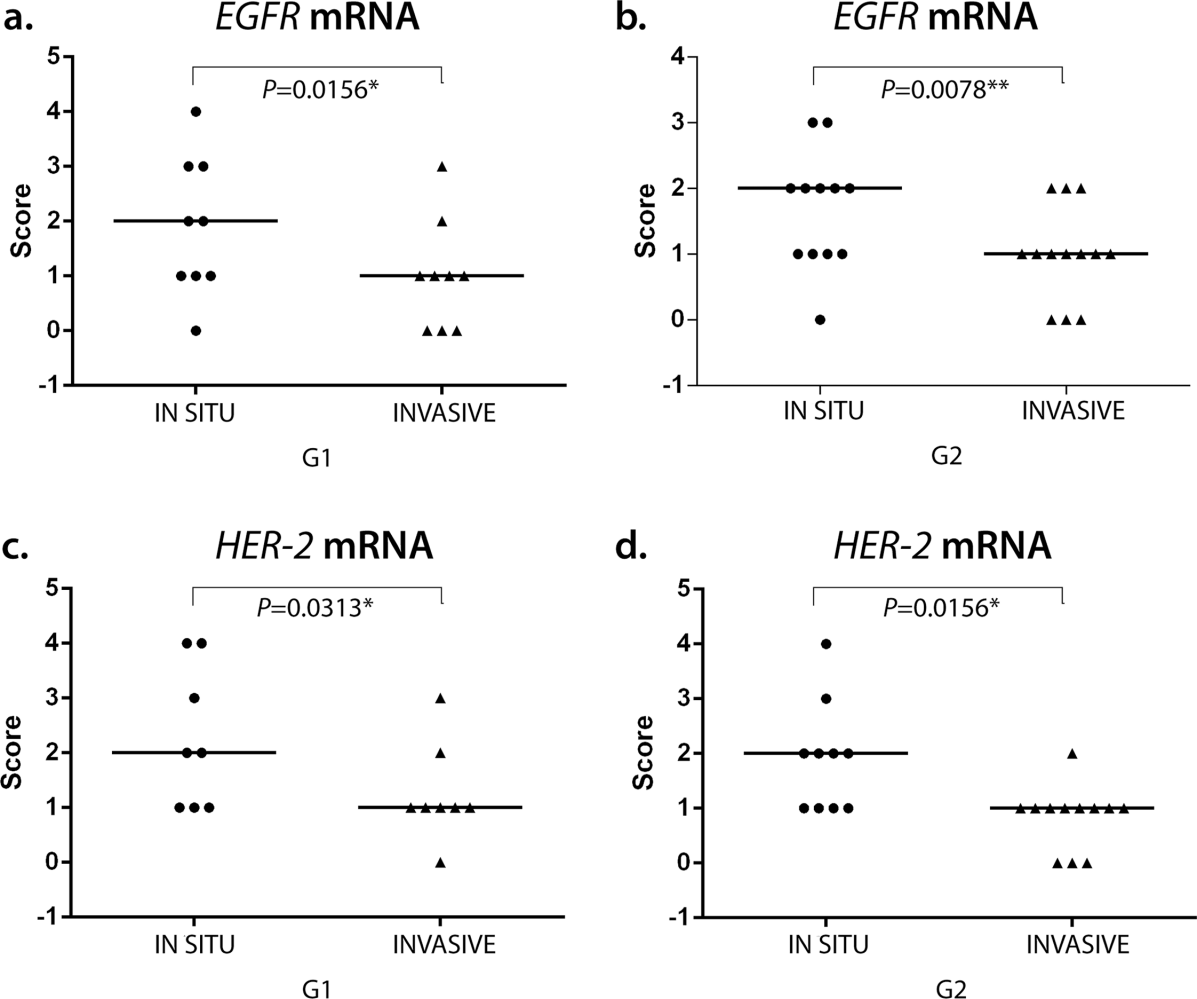
(A)(B) *EGFR* and (C)(D) *HER-2* mRNA expression in canine mammary tumours. Difference between *in situ* (IS) and invasive (IN) areas in versican low (G1) and high (G2) expression groups. Wilcoxon test. *p<0.05 ***p<0.001.

**Fig 3:**
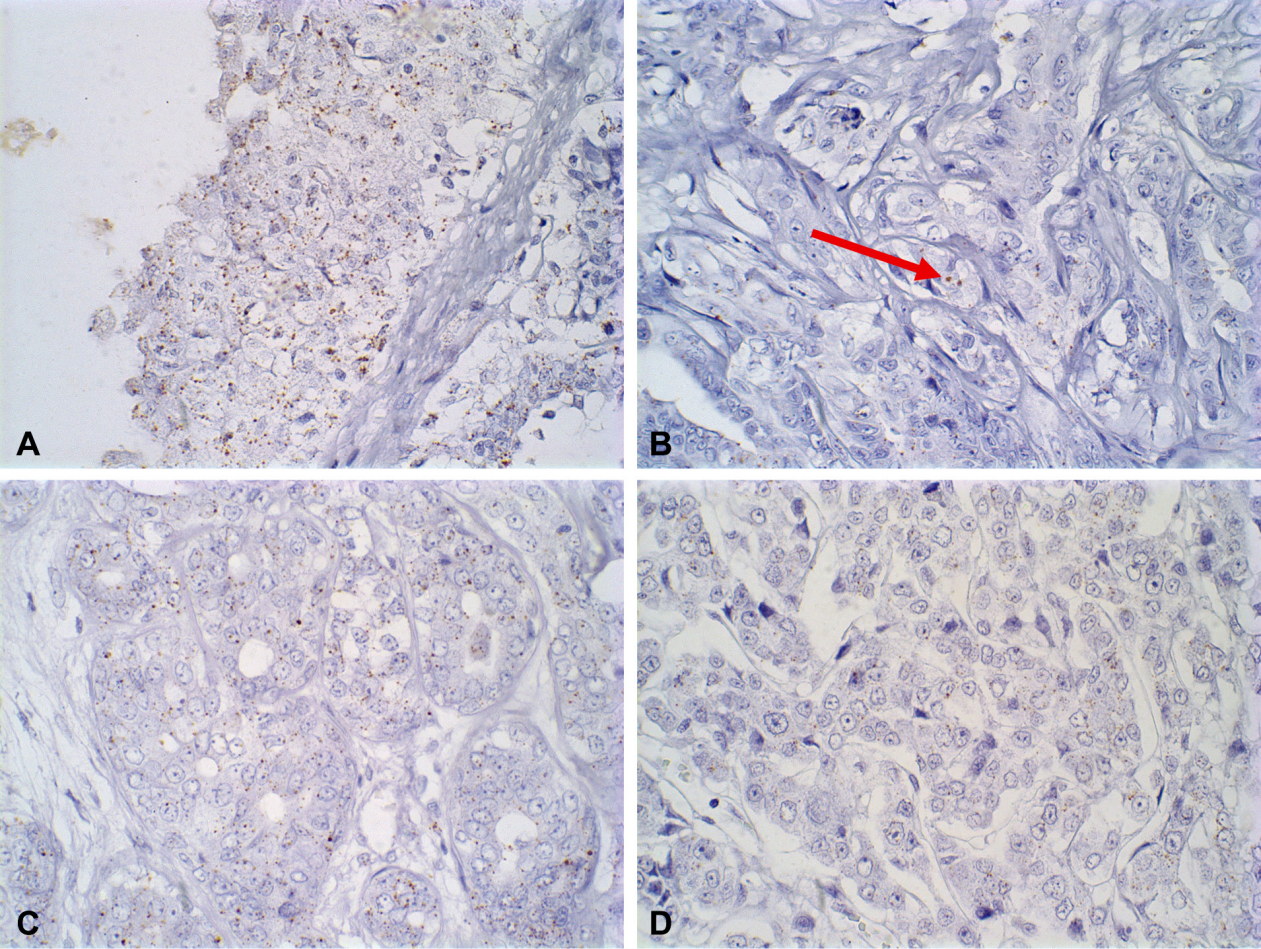
RNA-ISH analysis of *EGFR* gene expression in canine mammary tumours showing cytoplasmatic dots. (A) *In situ* carcinomatous areas showing RNA ISH score 3: in carcinomas in mixed tumours. (B) Invasive carcinomatous areas showing RNA ISH score 0 in carcinomas in mixed tumours (dots indicated by red arrow). (C) *In situ* carcinomatous areas showing RNA ISH score 2 in carcinosarcoma. (D) Invasive carcinomatous areas showing RNA ISH score 1 in carcinosarcoma (magnification x60).

**Fig 4:**
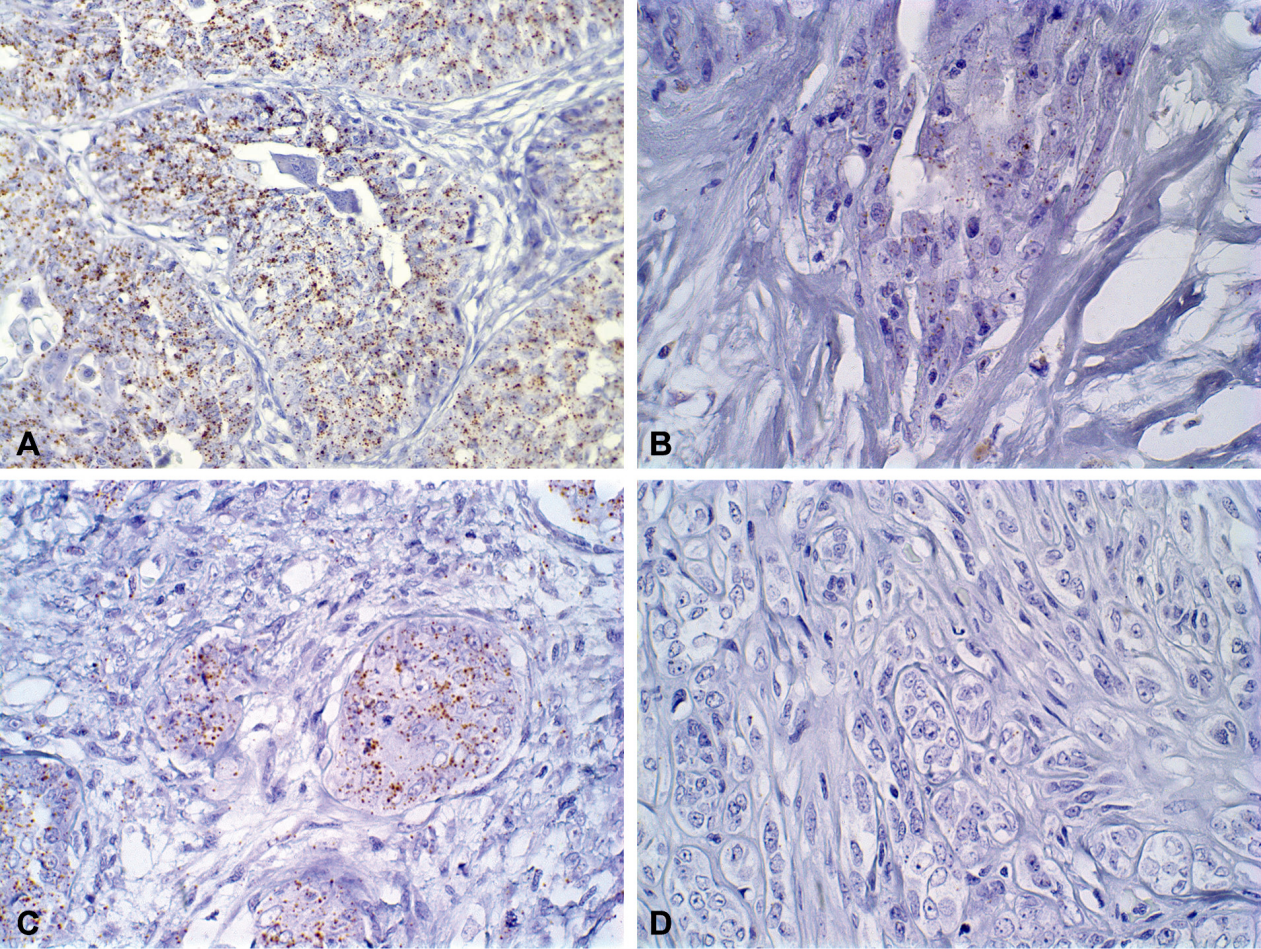
RNA-ISH analysis of *HER-2* gene expression in canine mammary tumours showing cytoplasmatic dots. (A) *In situ* carcinomatous areas showing RNA ISH score 4 in carcinomas in mixed tumours (magnification x40). (B) Invasive carcinomatous areas showing RNA ISH score 1 in carcinomas in mixed (magnification x60). (C) *In situ* carcinomatous areas showing RNA ISH score 3 in carcinosarcoma (magnification x60). (D) Invasive carcinomatous areas showing RNA ISH score 0 in carcinosarcoma (magnification x60).

ISH evaluation of *EGFR* mRNA expression showed a significant difference between *in situ* and invasive carcinomatous areas in both groups (low and high versican expression), the first showing more dots than the last (*P* = 0.0156 and *P* = 0.0078, respectively) (Figs 2A, 2B and 3). The same results were observed in *HER-2* mRNA expression, in which *in situ* areas showed higher staining than invasive areas in both groups (*P* = 0.0313 and *P* = 0.0156, respectively) (Figs 2C, 2D and 4). No statistical difference was observed between low and high versican expression groups.

The *EGFR* mRNA in *in situ* areas showed a relationship with histological grade (*P* = 0.0025, r = 0.432). In addition, pulmonary metastasis presented a positive correlation with *EGFR* mRNA expression in the invasive carcinomatous areas (*P* = 0.047, r = 0.375). Correlations were also observed between *HER-2* mRNA expression and lymph node metastasis in the *in situ* carcinomatous areas (*P*<0.0001, r = 0.381) and invasive carcinomatous areas (*P* = 0.017, r = 0.504).

### Expression of EGFR and HER-2 in carcinomatous areas

The *in situ* and invasive carcinomatous areas of neoplasms (CMTs and CSs) were analyzed individually. *In situ* carcinomatous cells displayed strong membrane EGFR expression (score 3+) (66.67%) and moderate membrane HER-2 expression (65%). Few cases (4.35% in both) exhibited strong membrane EGFR and HER-2 immunoreactivities (Table 2).

**Fig 5:**
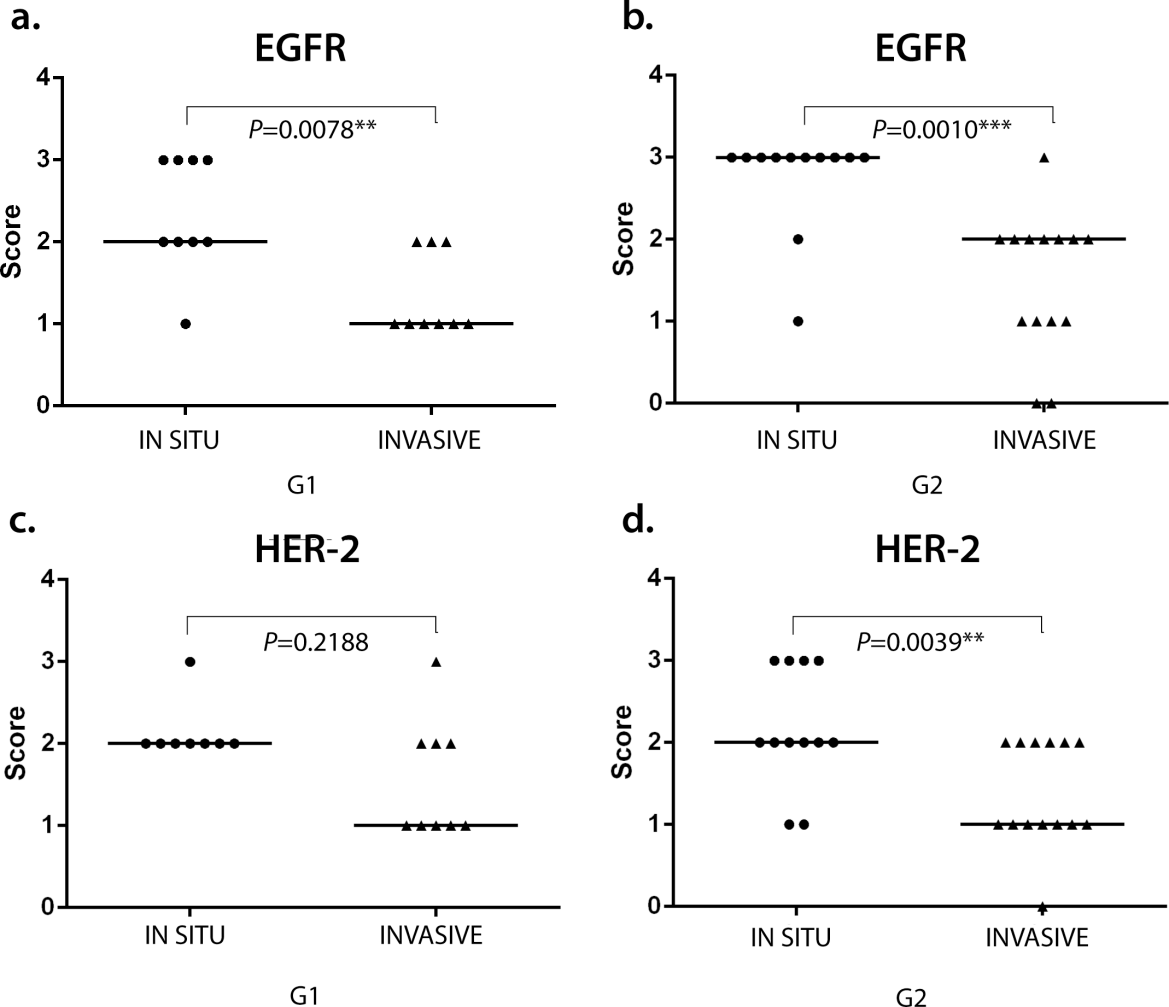
(A)(B) EGFR and (C)(D) HER-2 expression in canine mammary tumours. Difference between *in situ* (IS) and invasive (IN) areas in versican low (G1) and high (G2) expression groups. Wilcoxon test. *p<0.05 ***p<0.001.

Neoplasms with low versican expression showed greater EGFR immunostaining in the *in situ* areas than in invasive areas (*P* = 0.0078), even as the group presenting high versican expression displayed greater EGFR (*P* = 0.001) and HER-2 (*P* = 0.0039) staining in the *in situ* areas (Fig 5).

## Discussion

The search for new biomarkers and more effective therapeutic targets in cancer treatment warrants the interest in versican and its role in tumor progression. A better understanding of the relationship between versican and cell surface receptors in spontaneous tumors in canine mammary glands may contribute to identifying new tumor progression markers.

We have previously shown an increased versican expression in stroma adjacent to invasive areas in canine carcinomas in mixed mammary gland tumors [16]. These results suggested that other cell factors might be involved in activating this molecule and led us to investigate the interaction mechanisms between stromal versican and cell surface receptors in spontaneous mammary tumors in dogs. Previously, we evaluated the expression of tyrosine kinase receptors such as EGFR and HER-2 in benign mixed tumors, carcinomas in mixed tumors, and carcinosarcomas. Our results indicated that CSs exhibited a higher expression of HER-2, but not EGFR, in epithelial invasive cells in cases with a higher stromal versican expression. Here, we applied the ISH technique to determine whether mRNA transcripts of *EGFR* and *HER-2* oncogene in canine CMTs and CSs correlate with protein expression.

Versican coordinates complex signal pathways by interaction with EGF receptor. It had been demonstrated that overexpression of versican by G3 domain-containing EGF-like motifs enhanced breast cancer self-renewal through EGFR/AKT/GSK3β signaling, and increased resistance to chemotherapy [29]. However, different versican isoforms trigger different responses in cancer cells [30]. V2 isoform expression can inhibit tumor cell proliferation and metastasis through EMT modulation and by down-regulating EGFR signaling through ERK/GSK3β pathways [31, 32]. Other isoform of versican V3 can also regulate cell proliferation and migration by altering signaling pathways by interferring with CD44 and dimer EGFR/HER-2 [10].

Both the high and low versican expression groups presented higher *EGFR* and *HER-2* mRNA expression in *in situ* relative to invasive carcinomatous cells. Although, a difference between the groups was not observed, the interaction between cell surface receptors and versican cannot be discarded. Previous findings showing the relationship between HER-2 and versican expression in carcinomas in mixed tumors and carcinosarcomas support this hypothesis [16]. We suggest that overexpression of versican in peritumoral stroma can trigger signaling pathways, which would inhibit the transcription and translation of these receptors. However, the specific mechanisms by which this inhibition occurs remains unknown.

The mRNA expression of both EGFR and HER-2 receptors seems to be more intense in *in situ* carcinomatous areas than in invasive areas at the evaluated histologic types. The EGFR and HER-2 protein expression also showed an increased expression in *in situ* carcinomatous areas. Aguiar *et al*. demonstrated a similar distribution of EGFR and HER-2 expression between *in situ* and invasive carcinomas in human breast cancer [33]. Our findings support the participation of these molecules in early events in tumor progression.

Protein quantitation is the main target for detecting membrane receptor gene expression levels, but when immunohistochemistry shows background or the determination is complicated or not definitive, RNA detection is a good alternative [34]. Similar to mRNA results, immunohistochemical analysis revealed that EGFR and HER-2 were also overexpressed in *in situ* relative to invasive areas. These findings corroborate with previously described data [14, 27, 35–37].

Studies on tyrosine kinase mRNA expression in breast cancer have suggested both correlation and divergence between gene and protein expressions [38–41]. Divergence between molecules transcription and the translated proteins has already been described in breast cancer [40, 41]. Reis-Filho *et al*. demonstrated that most human metaplastic breast carcinomas, a histological subtype that presents histological similarities to canine mixed tumors, also overexpressed EGFR without gene amplification [41] and speculated that it might only reflect the maintenance of the basal-like/myoepithelial phenotype of these lesions. Viale *et al*., in a large randomized multicenter trial, showed lower concordance rates of protein and mRNA HER-2 assessments [42]. The analysis indicates that tumor heterogeneity and extent of DCIS and normal tissue components are not the likely causes of any differences between mRNA and protein assessment. In this study, no correlation was observed between EGFR and HER-2 mRNA and proteins expression evaluated in immunohistochemistry.

Öztürk *et al*. described that, in IHC HER-2 positive but ISH negative samples, the negativity could be explained by mRNA loss during fixation procedures [40]. However, there is a possibility that tumor cells slightly overproduce HER-2 protein without any observed increase in mRNA or DNA amplification. Furthermore, in the case where the mRNA and IHC signals are respectively positive and negativity, the oncoprotein might not be localized in the plasma membrane even though *HER-2* mRNA is overexpressed. In this study, we observed positive cytoplasmic staining in some tumor samples, but they were not considered as immuno-reactive because they did not demonstrate a distinct membrane staining. Comparative studies with a larger number of samples aimed at determining the association or relationship of cytoplasmic immunoreactivity and mRNA, and their distribution in tumor cells, will provide additional information and clarity on these issues.

Significant *EGFR* and *HER-2* mRNA, and protein expression in *in situ* carcinomatous sites relative to invasive areas suggest these molecules play a role during the early stages of tumor progression. However, further studies are needed to explain the biological differences that we observed from immunohistochemistry and *in situ* hybridization analysis. In addition, mRNA and versican expression do not correlate with each other. It is possible that other activation and signaling pathways are triggered by the interaction between versican in the extracellular matrix and neoplastic cells.

## References

[1] Hu M, Yao J, Carroll DK, Weremowicz S, Chen H, Carrasco D, et al Regulation of in situ to invasive breast carcinoma transition. Cancer Cell. 2008;13(5):394–406. 10.1016/j.ccr.2008.03.007 18455123 PMC3705908

[2] Theocharis AD, Skandalis SS, Neill T, Multhaupt HA, Hubo M, Frey H, et al Insights into the key roles of proteoglycans in breast cancer biology and translational medicine. Biochim Biophys Acta. 2015;1855(2):276–300. 10.1016/j.bbcan.2015.03.006 25829250 PMC4433619

[3] Nwabo Kamdje AH, Seke Etet PF, Vecchio L, Muller JM, Krampera M, Lukong KE. Signaling pathways in breast cancer: therapeutic targeting of the microenvironment. Cell Signal. 2014;26(12):2843–56. 10.1016/j.cellsig.2014.07.034 . 25093804

[4] Bertagnolli AC, Ferreira E, Dias EJ, Cassali GD. Canine mammary mixed tumours: immunohistochemical expressions of EGFR and HER-2. Aust Vet J. 2011;89(8):312–7. 10.1111/j.1751-0813.2011.00803.x . 24635633

[5] Ferreira E, Bertagnolli AC, Gobbi H, Cassali GD. HER-2 gene expression in atypical ductal hyperplasia associated with canine mammary carcinomas. Arquivo Brasileiro de Medicina Veterinária e Zootecnia. 2014;66:609–12. 10.1590/1678-41626212

[6] Appert-Collin A, Hubert P, Cremel G, Bennasroune A. Role of ErbB Receptors in Cancer Cell Migration and Invasion. Front Pharmacol. 2015;6:283 10.3389/fphar.2015.00283 26635612 PMC4657385

[7] Ricciardelli C, Sakko AJ, Ween MP, Russell DL, Horsfall DJ. The biological role and regulation of versican levels in cancer. Cancer Metastasis Rev. 2009;28(1–2):233–45. 10.1007/s10555-009-9182-y . 19160015

[8] Gorter A, Zijlmans HJ, van Gent H, Trimbos JB, Fleuren GJ, Jordanova ES. Versican expression is associated with tumor-infiltrating CD8-positive T cells and infiltration depth in cervical cancer. Mod Pathol. 2010;23(12):1605–15. 10.1038/modpathol.2010.154 . 20729814

[9] Wu Y, Chen L, Cao L, Sheng W, Yang BB. Overexpression of the C-terminal PG-M/versican domain impairs growth of tumor cells by intervening in the interaction between epidermal growth factor receptor and beta1-integrin. J Cell Sci. 2004;117(Pt 11):2227–37. 10.1242/jcs.01057 . 15126624

[10] Hernandez D, Miquel-Serra L, Docampo MJ, Marco-Ramell A, Cabrera J, Fabra A, et al V3 versican isoform alters the behavior of human melanoma cells by interfering with CD44/ErbB-dependent signaling. J Biol Chem. 2011;286(2):1475–85. 10.1074/jbc.M110.127522 21078678 PMC3020756

[11] Ween MP, Oehler MK, Ricciardelli C. Role of versican, hyaluronan and CD44 in ovarian cancer metastasis. Int J Mol Sci. 2011;12(2):1009–29. 10.3390/ijms12021009 21541039 PMC3083686

[12] Miquel-Serra L, Serra M, Hernandez D, Domenzain C, Docampo MJ, Rabanal RM, et al V3 versican isoform expression has a dual role in human melanoma tumor growth and metastasis. Lab Invest. 2006;86(9):889–901. 10.1038/labinvest.3700449 . 16847433

[13] Wu YJ, La Pierre DP, Wu J, Yee AJ, Yang BB. The interaction of versican with its binding partners. Cell Res. 2005;15(7):483–94. 10.1038/sj.cr.7290318 . 16045811

[14] Damasceno KA, Ferreira E, Estrela-Lima A, Bosco Y, Silva LP, Barros AL, et al Relationship between the expression of versican and EGFR, HER-2, HER-3 and CD44 in matrix-producing tumours in the canine mammary gland. Histol Histopathol. 2015:11705 10.14670/HH-11-705 . 26666308

[15] Cassali GD, Cavalheiro Bertagnolli A, Ferreira E, Araujo Damasceno K, de Oliveira Gamba C, Bonolo de Campos C. Canine mammary mixed tumours: a review. Vet Med Int. 2012;2012:274608 10.1155/2012/274608 23193497 PMC3485544

[16] Damasceno KA, Bertagnolli AC, Estrela-Lima A, Ribeiro LG, Rabelo BS, Campos CB, et al Versican expression in canine carcinomas in benign mixed tumours: is there an association with clinical pathological factors, invasion and overall survival? BMC Vet Res. 2012;8:195 10.1186/1746-6148-8-195 23082892 PMC3534148

[17] Misdorp W, Cotchin E, Hampe JF, Jabara AG, Von Sandersleben J. Canine malignant mammary tumors. 3. Special types of carcinomas, malignant mixed tumors. Vet Pathol. 1973;10(3):241–56. 10.1177/030098587301000307 . 4360454

[18] Lavalle GE, Bertagnolli AC, Tavares WL, Cassali GD. Cox-2 expression in canine mammary carcinomas: correlation with angiogenesis and overall survival. Vet Pathol. 2009;46(6):1275–80. 10.1354/vp.08-VP-0226-C-FL . 19605908

[19] Sassi F, Sarli G, Brunetti B, Morandi F, Benazzi C. Immunohistochemical characterization of mammary squamous cell carcinoma of the dog. J Vet Diagn Invest. 2008;20(6):766–73. 10.1177/104063870802000608 . 18987226

[20] Genelhu MC, Cardoso SV, Gobbi H, Cassali GD. A comparative study between mixed-type tumours from human salivary and canine mammary glands. BMC Cancer. 2007;7:218 10.1186/1471-2407-7-218 18045453 PMC2233636

[21] Owen LN, WHO. Classification of tumours in domestic animals First ed. World Health Organization: Geneva; 1980 p. 3–53.

[22] Misdorp W. Histological classification of the mammary tumors of the dog and the cat In: Series S, editor. WHO International Histological Classification Tumors of Domestic Animals. Washington: DC: AFIP; 1999 p. 59.

[23] Cassali GD, Ferreira E, Estrela-Lima A, De Nardi AB, Ghever C, et al Consensus for the Diagnosis, Prognosis and Treatment of Canine Mammary Tumors—2013. Braz J Vet Pathol. 2014;7:38–69.

[24] Elston CW, Ellis IO. Assessment of histological grade In: Elston CW, Ellis IO, editors. The breast. 13. Churchill Livingstone, Edinburgh: New York; 1998 p. 356–84.

[25] Erdelyi I, van Asten AJ, van Dijk JE, Nederbragt H. Expression of versican in relation to chondrogenesis-related extracellular matrix components in canine mammary tumors. Histochem Cell Biol. 2005;124(2):139–49. 10.1007/s00418-005-0007-y . 16088379

[26] Skandalis SS, Labropoulou VT, Ravazoula P, Likaki-Karatza E, Dobra K, Kalofonos HP, et al Versican but not decorin accumulation is related to malignancy in mammographically detected high density and malignant-appearing microcalcifications in non-palpable breast carcinomas. BMC Cancer. 2011;11:314 10.1186/1471-2407-11-314 21791066 PMC3199864

[27] Wolff AC, Hammond ME, Hicks DG, Dowsett M, McShane LM, Allison KH, et al Recommendations for human epidermal growth factor receptor 2 testing in breast cancer: American Society of Clinical Oncology/College of American Pathologists clinical practice guideline update. J Clin Oncol. 2013;31(31):3997–4013. 10.1200/JCO.2013.50.9984. . 24101045

[28] Alves MR, Carneiro FC, Lavorato-Rocha AM, da Costa WH, da Cunha IW, de Cassio Zequi S, et al Mutational status of VHL gene and its clinical importance in renal clear cell carcinoma. Virchows Arch. 2014;465(3):321–30. 10.1007/s00428-014-1629-z . 25027579

[29] Du WW, Yang W, Yee AJ. Roles of versican in cancer biology—tumorigenesis, progression and metastasis. Histol Histopathol. 2013;28(6):701–13. . 23519970 10.14670/HH-28.701

[30] Kischel P, Waltregny D, Dumont B, Turtoi A, Greffe Y, Kirsch S, et al Versican overexpression in human breast cancer lesions: known and new isoforms for stromal tumor targeting. Int J Cancer. 2010;126(3):640–50. 10.1002/ijc.24812 . 19662655

[31] Lee HC, Su MY, Lo HC, Wu CC, Hu JR, Lo DM, et al Cancer metastasis and EGFR signaling is suppressed by amiodarone-induced versican V2. Oncotarget. 2015;6(40):42976–87. 10.18632/oncotarget.5621 26515726 PMC4767485

[32] Sheng W, Wang G, Wang Y, Liang J, Wen J, Zheng PS, et al The roles of versican V1 and V2 isoforms in cell proliferation and apoptosis. Mol Biol Cell. 2005;16(3):1330–40. 10.1091/mbc.E04-04-0295 15635104 PMC551496

[33] Aguiar FN, Mendes HN, Bacchi CE, Carvalho FM. Comparison of nuclear grade and immunohistochemical features in situ and invasive components of ductal carcinoma of breast. Rev Bras Ginecol Obstet. 2013;35(3):97–102. . 23538467 10.1590/s0100-72032013000300002

[34] Alba J, Gutierrez J, Coupe VM, Fernandez B, Vazquez-Boquete A, Alba J, et al HER2 status determination using RNA-ISH—a rapid and simple technique showing high correlation with FISH and IHC in 141 cases of breast cancer. Histol Histopathol. 2012;27(8):1021–7. . 22763874 10.14670/HH-27.1021

[35] Ricciardelli C, Russell DL, Ween MP, Mayne K, Suwiwat S, Byers S, et al Formation of hyaluronan- and versican-rich pericellular matrix by prostate cancer cells promotes cell motility. J Biol Chem. 2007;282(14):10814–25. 10.1074/jbc.M606991200 . 17293599

[36] Suwiwat S, Ricciardelli C, Tammi R, Tammi M, Auvinen P, Kosma VM, et al Expression of extracellular matrix components versican, chondroitin sulfate, tenascin, and hyaluronan, and their association with disease outcome in node-negative breast cancer. Clin Cancer Res. 2004;10(7):2491–8. 10.1158/1078-0432.CCR-03-0146 . 15073129

[37] Yee AJ, Akens M, Yang BL, Finkelstein J, Zheng PS, Deng Z, et al The effect of versican G3 domain on local breast cancer invasiveness and bony metastasis. Breast Cancer Res. 2007;9(4):R47 10.1186/bcr1751 17662123 PMC2206723

[38] Bhargava R, Gerald WL, Li AR, Pan Q, Lal P, Ladanyi M, et al EGFR gene amplification in breast cancer: correlation with epidermal growth factor receptor mRNA and protein expression and HER-2 status and absence of EGFR-activating mutations. Mod Pathol. 2005;18(8):1027–33. 10.1038/modpathol.3800438 . 15920544

[39] Latta EK, Tjan S, Parkes RK, O'Malley FP. The role of HER2/neu overexpression/amplification in the progression of ductal carcinoma in situ to invasive carcinoma of the breast. Mod Pathol. 2002;15(12):1318–25. 10.1097/01.MP.0000038462.62634.B1 . 12481013

[40] Ozturk M, Bolkent S, Yilmazer S, Kaner G, Unal H. Detection of c-erbB-2 mRNAs using dig-labelled oligonucleotide probe with in situ hybridisation in human breast carcinoma: comparison with immunohistochemical results. Anal Cell Pathol. 1998;16(4):201–9. 10.1155/1998/180738 9762367 PMC4617574

[41] Reis-Filho JS, Milanezi F, Carvalho S, Simpson PT, Steele D, Savage K, et al Metaplastic breast carcinomas exhibit EGFR, but not HER2, gene amplification and overexpression: immunohistochemical and chromogenic in situ hybridization analysis. Breast Cancer Res. 2005;7(6):R1028–35. 10.1186/bcr1341 16280056 PMC1410747

[42] Viale G, Slaets L, de Snoo FA, Bogaerts J, Russo L, Van't Veer L, et al Discordant assessment of tumor biomarkers by histopathological and molecular assays in the EORTC randomized controlled 10041/BIG 03–04 MINDACT trial breast cancer: Intratumoral heterogeneity and DCIS or normal tissue components are unlikely to be the cause of discordance. Breast Cancer Res Treat. 2016;155(3):463–9. 10.1007/s10549-016-3690-6 26820652 PMC4764628

